# Exploring Biomedical Waste Management Practices Among Healthcare Professionals: A Study From a Tertiary Care Teaching Hospital in Eastern India

**DOI:** 10.7759/cureus.61823

**Published:** 2024-06-06

**Authors:** Mukunda C Sahoo, Jawahar S K Pillai, Biswajeevan Sahoo

**Affiliations:** 1 Hospital Administration, All India Institute of Medical Sciences, Bhubaneswar, Bhubaneswar, IND

**Keywords:** knowledge and practices, behavioral interventions, quality improvement and patient safety, hcw training, biomedical waste management

## Abstract

Context: The generation of biomedical waste (BMW) in hospitals presents a significant hazard to both healthcare workers (HCWs) and the environment. The management of BMW is a challenge regarding inappropriate behavior among HCWs, leading to improper segregation of the BMW, which deserves attention. The indiscriminate BMW management issue in India has attracted the attention of the highest judicial bodies. The rise in the incidence of needle stick injuries is a severe threat to waste handlers and is mainly due to improper segregation practices.

Aim: This study aimed to identify the challenges in BMW management in the institute and develop a strategy to improve the knowledge and practices of healthcare professionals (HCPs) in BMW management.

Methodology: A process-based intervention was developed and implemented that involved facility inspection rounds, focused group discussions with HCWs, preparation of information, education, and communication (IEC) materials, signage, staff training, and improved infrastructure and supplies for waste collection. A questionnaire-based evaluation of the staff’s knowledge of BMW management was conducted, and the impact of the intervention was assessed from the hospital infection control audit reports.

Results: Multiple reasons for poor compliance with BMW segregation practices were identified, and it improved from 57% to 91% with interventions. A significant difference was noted in the knowledge level of staff before and after the interventions. Post-intervention score was highest among the nurses (98.5%), followed by sanitation staff (92.7%), doctors (89.25%), and paramedics (81.7%).

Conclusions: Incorrect segregation practices of BMW and incidents of occupational exposure to blood and body fluids are reduced with interventions in the study. Robust supply chain management with regular training of staff is vital to ensure compliance in BMW management.

## Introduction

The generation of biomedical waste (BMW) in hospitals presents a significant hazard to healthcare workers (HCWs) and the environment. The indiscriminate BMW management issue in India has attracted the attention of the highest judicial bodies. From time to time, the Honourable Supreme Court of India and Apex Courts issued instructions regarding managing BMW. In some of the prestigious institutes of the country, gross violations and non-compliance with biomedical rules were found in the past and have been pulled up by the National Green Tribunal (NGT) for failing to comply with the BMW rules. Under the Environment (Protection) Act of 1986, the Bio-Medical Waste Management Rules of 2016 came into force on March 28, 2016 [1] in supersession of the Bio-Medical Waste (Management and Handling) Rules of 1998. Thus, the effective management of BMW is a legal necessity and a social responsibility. BMW harbors pathogens and infectious agents. Incorrect segregation practices of BMW aid in spreading infections and make HCWs vulnerable to occupational exposure to infectious wastes. There is a risk of accidents and injuries to the hospital's HCWs, patients, and visitors.

Furthermore, hospitals are subjected to regulations governing the management of BMW. Compliance with these regulations is a collective responsibility of all HCWs to ensure that BMWs do not contaminate our environment. "Let the waste of the sick not contaminate the lives of the healthy" is a phrase [2] that refers to the importance of preventing the spread of infection to protect patients and HCWs from disease.

Adequate resource allocation and proper infrastructure for handling BMW are essential. At the same time, designing an educational intervention for various cadres of healthcare professionals (HCPs) and infrastructural development for waste management is also necessary. At present, there needs to be more evidence-based training programs in the domain of BMW and disposal.

The study institution is a 1,000-bed public-sector tertiary-level teaching hospital in eastern India. It has a daily outpatient department (OPD) footfall of about 4,000 patients, 100 admissions, 200 patients in the emergency department, and about 50 major surgeries per day. About 4,000 staff are working in all three shifts.

The increases in incidents of occupational exposure to blood and body fluids due to incorrect BMW segregation practices were noted in the reports from the Hospital Infection Control Unit. This study is an effort to develop a strategy in BMW management by incorporating scientific and evidence-based knowledge to promote personal, environmental, and patient safety.

The present study aimed to identify the challenges in BMW management in the institute and develop a strategy to improve the knowledge and practices of HCPs in BMW management.

## Materials and methods

The study was conducted at All India Institute of Medical Sciences (AIIMS), Bhubaneswar, in Bhubaneswar, India, in two phases. The duration of the study was 12 months. Phase 1 is observational and aims to understand the problem statement. It was conducted over three months. Phase 2 of the study was pre-post intervention in nature and was conducted over nine months. This phase included interventions like infrastructural development and training of HCWs, followed by a questionnaire-based evaluation of the HCWs on their knowledge of BMW management and monitoring of the actual practices of the HCWs dealing with BMW in the study setting.

AIIMS, Bhubaneswar, issued approval (reference no. IMF/30/2018).

Phase 1

To begin with, a team consisting of faculty members and residents from the Department of Hospital Administration, Nurses, and Sanitary Inspectors conducted inspections of the areas of BMW generation, which included OPD procedure rooms, wards, intensive care units, operation theatres, dialysis units, emergency departments, labor room, and laboratories. The findings were consolidated. Focused group discussions (FGDs) were conducted with all categories of staff in the patient care areas to understand the reasons for the incorrect waste segregation practices and other issues. Process maps for waste generation were prepared. Quality tools like five-whys, fishbone diagrams, and checklists were used. An action plan was developed, which consisted of the following points: a) improvement of facilities for BMW collection in various patient care areas, b) ensuring timely and adequate supplies of color-coded waste collection bags, c) a separate waste handler team was created to collect waste from the whole hospital as per schedule, d) development of pictographic IECs (posters, handbooks, and short videos), e) training needs assessment of the staff and department with the highest non-compliance in BMW management practices, f) periodic training of the staff, and g) monitoring of the BMW segregation in the patient care areas by the hospital infection control team.

Phase 2

The interventions consisted of improving the infrastructure and training of HCWs. The areas of BMW generation were identified in ICUs, wards, OT, OPDs, daycare, trauma, dialysis, and others. Signages were installed to identify the areas. Meetings were conducted with the procurement division for regular supply of color-coded waste collection bags and prevention of stock-outs. Availability of foot-operated dustbins and bags of different volumes was ensured in procedure rooms and other areas by an equitable distribution of the bins and bags in each department. Small-volume dustbins were put in the treatment trolleys that could be taken near the procedure areas or bedside. A standard operating procedure (SOP) was prepared and made available to all staff.

**Figure 1:**
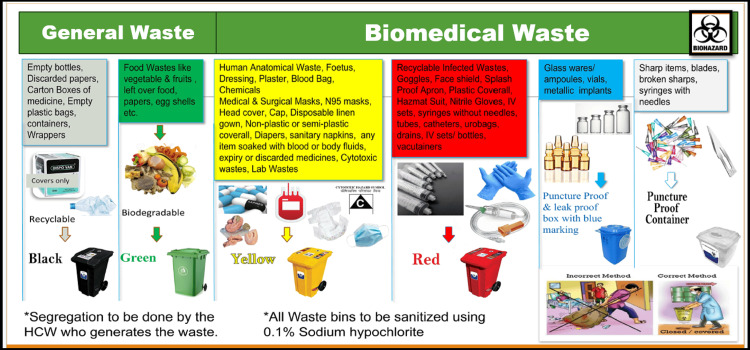
information, education, and communication (IEC) materials for waste segregation

Training consisted of one-to-one training in the patient care areas and common training. Based on the Guidelines for the Management of Healthcare Waste as per the Biomedical Waste Management Rules of 2016 [1], Government of India, pictographic signages and IEC were developed and installed in all the patient care areas (Figure 1). Pocket-sized IEC cards were distributed among the HCWs of the hospital during the hospital rounds for a quick read, which can be kept in the apron pocket. Staff were encouraged to refer to the IECs for the segregation of BMW generated at the time of any medical procedure.

**Figure 2:**
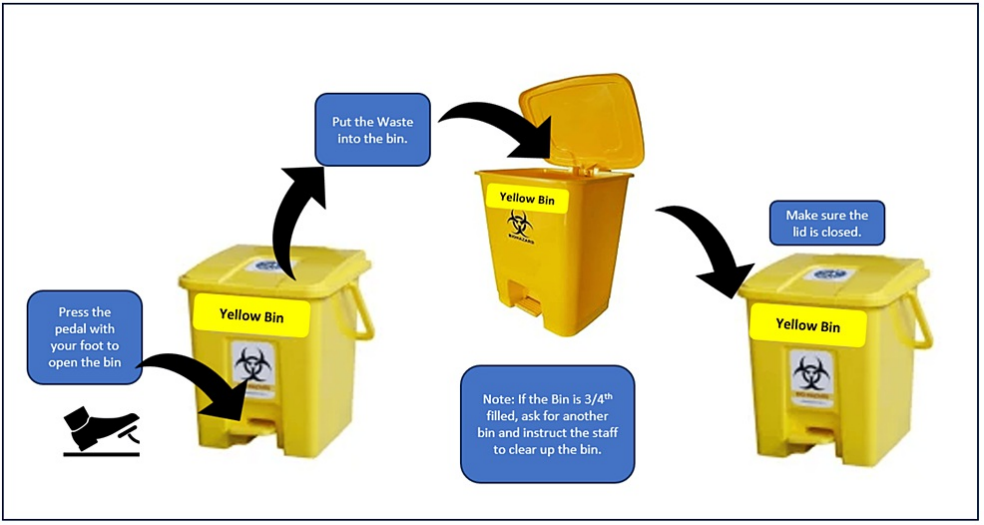
information, education, and communication (IEC) material for the correct method of using a pedal-operated dust bin

A practical demonstration of the usage of different types of waste bins available in the hospital was demonstrated in the training (Figure 2).

Small video clips were circulated in social media groups of HCWs.

A questionnaire (Table 1) validated by the domain experts was used to assess HCWs' knowledge of BMW management. Each question carried a score of 1 for correct response and 0 for incorrect response. It was translated into the regional language for the sanitation staff. The variables included the category of the staff, gender of the participant, and awareness of the staff about the BMW management, which are statutory guidelines, reporting of accidents to authority for regulating and monitoring of BMWs, categorization of BMW, color coding of collection bags and dustbins, liquid waste management, labeling of the bags, cytotoxic wastes, steps in handling BMWs, occupational safety, immunization of the HCWs, and the responsibility of the HCWs in managing the BMWs. 

**Table 1:** Questionnaire for assessment

Sl, No	Question
1	The last major amendment in biomedical waste management was done in 2016. A) True, B) False
2	The wastes generated during the diagnosis, treatment, and immunization of humans and animals are considered biomedical wastes. A) True, B) False
3	Biomedical wastes can be in both solid and liquid forms. A) True, B) False
4	The correct process of biomedical waste is: A) generation > segregation > transportation > collection > storage > treatment and disposal; B) generation > segregation > collection > storage > transportation > treatment and disposal; C) transportation > generation > segregation > treatment > collection > storage; D) generation > collection > segregation > storage > transportation > treatment and disposal.
5	The management of biomedical waste in India is carried out by: A) We can manage on our own at the hospital campus by burning. B) State government. C) Agencies authorized by the Pollution Control Board. D) Ministry of Health and Family Welfare.
6	The biomedical waste from the hospital is transported to the common treatment facility. It can be monitored through online tracking in the mobile device. A) True, the waste packets are bar-coded and therefore can be tracked. B) False, it is not possible to track all bags.
7	How many categories of waste are generated as per the Biomedical Waste Management Rules of 2016? A) 6, B) 4, C) 10, D) 8
8	Select the correct color coding of the waste collection bins specific to biomedical wastes: A) Yellow bin, red bin, and puncture-proof container. B) Red bin, green bin, and puncture-proof container. C) Blue bin, green bin, and puncture-proof container. D) Blue bin, green bin, yellow bin, and puncture-proof container.
9	Can food waste be considered as biomedical waste? A) Yes, if it is used by the COVID-19 patient. B) No, it is a general waste.
10	Objects that are capable of causing punctures or cuts, that may have been exposed to blood or body fluids including scalpels, needles, glass ampoules, test tubes, and slides, are considered biomedical waste. How should these objects be disposed of? A) Ask the nurse or any other staff to dispose. B) Put it in a puncture-proof container.
11	Which category of waste is usually microwaved before handing over to the authorized vendor? A) Yellow. B) Red. C) Black. D) No waste is microwaved.
12	Labeling of the BMW waste bag is essential to track the sources of the waste. A) True, B) False
13	Cytotoxic waste should be put in a yellow-color bag labeled with the symbol "C" for cytotoxic waste. A) True, B) False
14	Which steps should be followed after exposure to infected blood/body fluid and contaminated sharps? A) Exposed parts are to be immediately washed with soap and water. B) Inform the infection control nurse. C) Follow the instructions and start post-exposure prophylaxis if advised by the doctor. D) All of the above.
15	One of the important parts of biomedical waste management is the segregation at the source. A) True, B) False
16	Liquid biomedical waste cannot be disposed of as it is, but it should be mixed with an appropriate volume of freshly prepared sodium hypochlorite solution. A) True, B) False
17	Blood bags are to be disposed of in which color bin? A) Red, B) yellow, C) blue PPC, D) puncture-proof container
18	Catheters, urine bags, and syringes without needles should be put in a A) red, B) yellow, C) blue PPC, or D) puncture-proof container.
19	One of the common causes of needle stick injury among waste handlers is due to incorrect waste segregation practices. A) True, B) False
20	Biomedical wastes should be removed from the area of generation within 48 hours. A) True, B) False
21	Biomedical waste should be removed whenever the bin is two-thirds full and should not be over-packed. A) True, B) False
22	The transportation of biomedical waste must be done in closed containers to prevent leakage and spillover of the waste and also prevent it from stray animals. A) True, B) False
23	Biomedical waste management is the responsibility of the generator of the waste. This means that all healthcare workers in the hospital are equally responsible for BMW management. A) True, B) False
24	We must report any accidents that happen in the process of BMW management to the concerned authority. A) True, B) False
25	All healthcare workers should get immunized for hepatitis B. A) True, B) False
26	All healthcare workers in the hospital should be trained in BMW Management irrespective of nature as they must know the correct method and can prevent incorrect practices. A) True, B) False

Official intimations were sent to each department about the proposed training sessions. A training calendar was prepared for a period of two months. Individual departments nominated staff to attend the training sessions. 

Convenient sampling method was used and a total of 352 HCWs participated in the training program that included doctors (60), nurses (158), paramedical (44), and sanitation staff (90). The pre-intervention data were collected from the participants at the beginning of the training sessions. A total of 12 training sessions were held. The post-intervention data were collected by the study team from individual participants after a period of two months after he/she has attended the training session. 

The findings were analyzed using the paired t-test. The training sessions consisted of didactic lectures, group exercises, and quizzes on the Guidelines and Practices in Biomedical Waste Management. It also aimed to create a sense of responsibility for appropriate waste segregation among the study participants. The hazardous impact of the wrong practices on the waste handlers and other stakeholders was shown to the participants. Case stories of the staff who had been the victims of NSIs and had to be take the post-exposure prophylactic drugs were discussed in the session ensuring anonymity of the identity of the staff.

Monitoring

The BMW segregation practices of the HCWs are physically audited at the point of generation, its collection areas, and in the waste collection yard. Monthly reports from the hospital infection control team for the duration of the study (12 months) were used to assess the impact of the interventions.

## Results

Findings from inspection rounds and FGDs

The research identified the reasons for poor segregation practices. These included lack of clarity in segregation methods among the staff, carelessness among staff due to ignorance, lack of commitment from the senior doctors about the practice of BMW management by their junior colleagues, lack of awareness about regulatory guidelines among the staff, inadequate training, time constraints due to the high workload, high turnover among the staff, irregular supplies of waste collection bags, location or placement of dustbins unknown to the staff, and inadequate monitoring and enforcement of waste management policies in the hospital.

**Figure 3:**
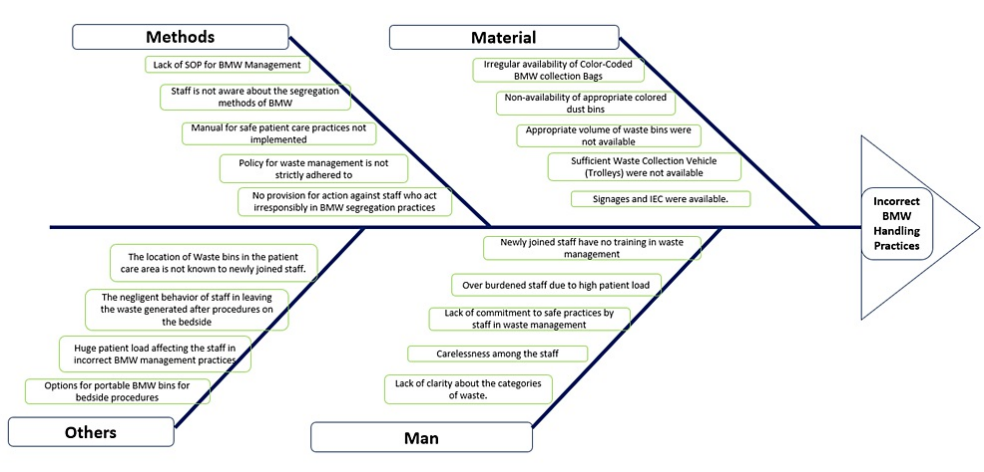
Fish bone diagram for incorrect BMW practices

The most common reason for incorrect BMW segregation practices mentioned by the staff was the non-availability of the color-coded waste bags. Due to this reason, the sanitation staff were compelled to put different colored waste bags in the dust bin, thus violating the guidelines. Other reasons are listed in the fish bone diagram (Figure 3). Another important challenge identified is the staff turnover. Since the study setting is a medical college, a regular turnover of staff (residents, interns, and nurses) is observed. 

Findings from the questionnaire-based evaluation of the knowledge of HCWs

**Figure 4:**
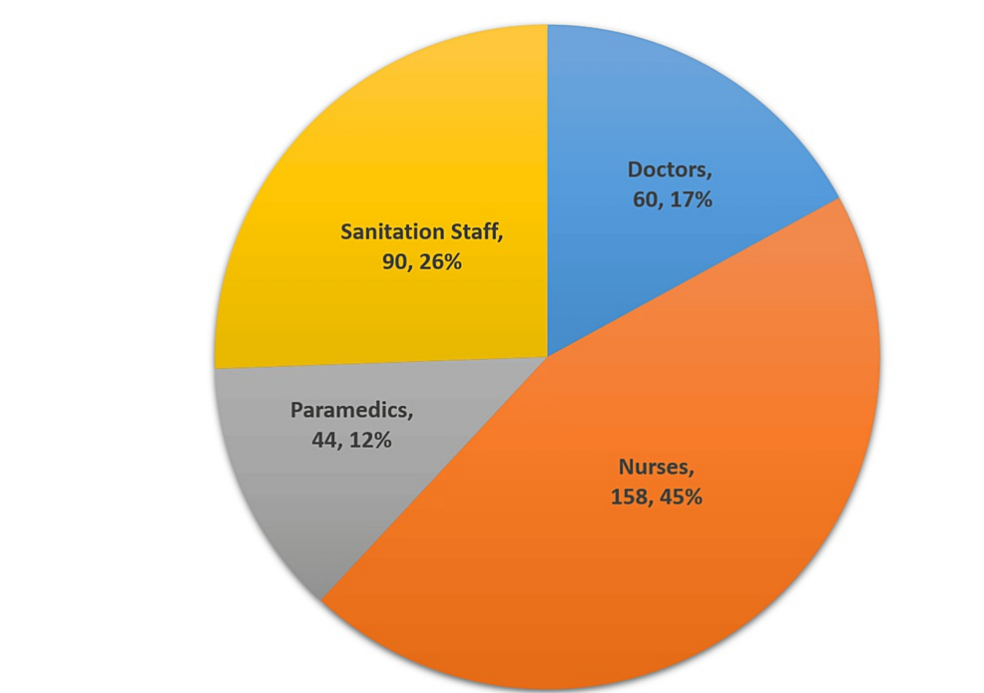
Category of participants in the questionnaire-based assessment

Out of the total participants in the study (Figure 4), 45% were nurses, 26% were sanitation staff, 17% were doctors, and 12% were paramedics.

The 215 (61%) participants were females, while the 137 (39%) participants were males. 

**Figure 5:**
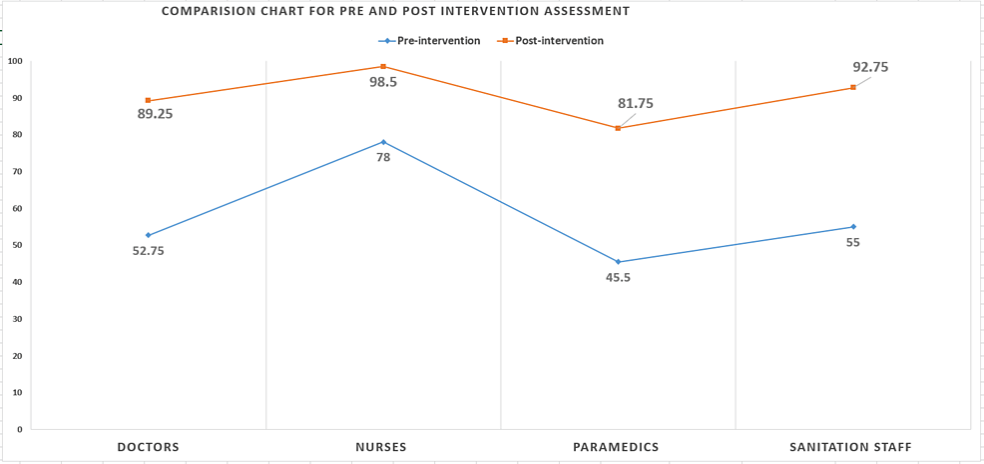
Comparison chart for the pre- and post-intervention mean scores Y-axis: mean score in percentage, X-axis: categories of the study participants

In the pre-intervention evaluation (Figure 5), it was found that the nurses had the highest level (mean score 78%) of the knowledge of BMW management practices, while the paramedics had the lowest (mean score 45.5%). The mean score for the doctors increased from 52.75 % to 89.25%; for the nurses, it increased from 78% to 98.5%; for the paramedics, it increased from 45.5% to 81.75%; and for the sanitation staff, it increased from 55% to 92.75%. 

Hypothesis testing was done with the null hypothesis being no difference in the knowledge level of BMW management practices among HCWs assessed in the pre- and post-intervention data and the alternate hypothesis of a significant difference between the pre- and post-intervention data. A paired t-test was performed to analyze the mean scores in the pre-intervention (μ1 = 0.68) and post-intervention data (μ2 = 0.96). The t-value was 51.69 and the p-value was *<0*.00001 at a significance level of 0.05. The result is significant at p < 0.05, thereby accepting the alternate hypothesis of the significant difference between the knowledge level of staff before and after the interventions.

Impact assessment

**Figure 6:**
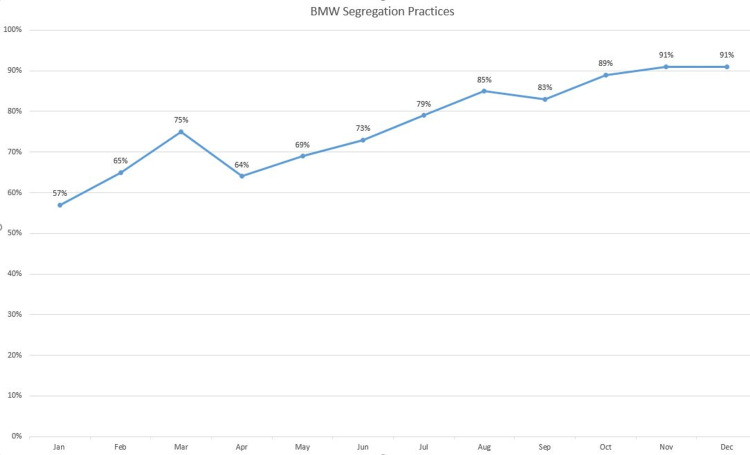
Biomedical waste (BMW) segregation practices' audit report for the study period

Ensuring the regular supply of BMW bags and easy accessibility of waste bins helped improve segregation practices. The Hospital Infection Control team reports showed an overall improvement in BMW segregation practices from 57% to 91% (Figure 6).

Similarly, the incidence of occupational exposure, such as needle stick injuries due to improper sharp management, showed a marked reduction from 12 Incidents at the beginning of the study period to 3 at the end of the study.

## Discussion

It is evident from various studies that managing healthcare waste is a significant challenge in India and other parts of the world. Continuous training programs make aspects like skill development and awareness generation possible. However, behavioral modification is challenging as it is multifactorial.

The training sessions helped create a sense of responsibility among the HCWs by creating awareness about the consequences of needle stick injuries among the waste handlers, primarily due to improper BMW management practices. 

Singh et al. [3] conducted a study on training HCPs about the various aspects of BMW management and found significant improvement in the knowledge of HCWs with structured training programs. A similar result was evident in this study. 

Mathur et al. [4] did a cross-sectional study to assess the knowledge and attitude of HCWs regarding BMW handling and found that the doctors, nurses, and technicians had better understanding than the housekeeping staff. On the contrary, in this study, the segregation practices among the sanitation/housekeeping staff were at par with the doctors. The improvement in BMW practices was found to be the highest among the sanitation staff.

In a similar study done in an African hospital, Mugabi et al. [5] identified that the lack of knowledge about the dangers of improper waste management by HCWs is a significant obstacle to ensuring correct practices. They concluded that the ongoing training for HCWs is vital with more attention to regulatory requirements and opined that HCWs should be involved in developing institutional policies for BMW management to enhance compliance.

Kapoor et al. [6] systematically reviewed knowledge and awareness regarding BMW in dental teaching institutions. They concluded that the understanding of color coding-based segregation is poor among HCWs. The present study also identified the same and thus gave maximum importance to building up knowledge of the appropriate segregation practices. 

During the training sessions, it was noted that female HCWs were more serious about the hazards of BMW and the safety of the staff from occupational exposure to infectious waste. 

Raghuvanshi et al. [7] found that 80% of doctors had knowledge of BMW, but only 41% were practicing correctly. The study mentioned that the doctors and nursing officers in the study hospital had poor BMW segregation practices, which were attributed to the workload on staff and the absence of dustbins in the waste generation area.

Gonibeedu et al. [8] conducted a cross-sectional study among the primary health centers in a district. They assessed the knowledge, attitude, and practice of BMW management through observation, staff interviews, and record review on a predesigned questionnaire. The knowledge level was higher than that of attitude and practice, and therefore, it was concluded that there is a need for repeated training with strict supervision. 

Njagi A [9] used a self-administered questionnaire to identify the gaps with a focus on their knowledge, attitude, and practice among clinicians, nurses, laboratory technologists, and hospital attendants in hospitals in Kenya. They also found that safety in BMW management is not included in the academic curriculum of the HCWs, and they acquire this through on-the-job training, seminars, and talks. The strategy was similar to the present study as the training improved segregation practices and reduced injuries from healthcare waste. 

Pandey et al. [10] mentioned that although the awareness of BMW practices was high among the HCWs, the segregation practices were poor. Thus, training in smaller groups focusing on behavior is necessary at regular intervals. 

In their review article, Dutta et al. [11] emphasized the need for committed collective teamwork by all categories of HCWs, along with financial support and adequate infrastructure for the effective management of hospital wastes. Similar challenges were identified in the present study.

Kumar et al. [12] conducted a checklist-based audit of the aspects of BMW management at the point of generation. They emphasized the mutilation and disinfection of recyclable waste in the treatment rooms, especially by the resident doctors.

Similarly, Ramalingam et al. [13] conducted a checklist-based assessment of BMW management in operation theaters, wards, ICUs, and other areas of a tertiary care hospital. They assessed the condition of the waste containers, segregation practices, and mutilation of recyclable wastes. They identified that the segregation of waste carries the utmost importance, and the mutilation of recyclable waste in the emergency department and the wards was significantly lower than that of OTs and intensive care units.

Bansod et al. [14] conducted a systematic review study on the importance of BMW management. They highlighted the poor education of waste handlers about BMW and emphasized that the staff needs to be aware of the dangers of BMW waste and avoid accidents due to improper handling. 

Devi et al. [15] studied the factors that affected the performance of healthcare facilities and concluded that regular training and better infrastructure can improve BMW management practices at healthcare centers. It was also evident in the present study.

Padmapriya [16] mentions the risk of exposure to the BMW and getting infected with pathogens, as all levels of patient care include diagnostics and therapeutic areas. Although the regulatory framework exists, improper segregation is still evident in most studies. It is high time for every HCW to be serious about improving their and colleagues’ practices in handling BMWs in their respective healthcare settings.

Strengths of the study

In the present study, a deep dive was done to understand the reasons for non-compliance by the HCWs, and necessary interventions were initiated. Subsequently, the impact was also assessed. The two-phase approach provided a comprehensive way to bring improvement in the system. FGDs provided insight into the problem faced by the staff and therefore provided direction in the action plan. The segregation practices among the HCWs showed significant improvement in waste management practices, including infrastructure and supply chain improvement, along with multimodal training as an effective intervention. Sensitizing the HCWs about the outcome due to incorrect practices brought a behavioral change among them. The incidence of occupational exposures like needle stick injury among waste handlers was reduced. 

Limitations of the study

The study was conducted in a single large tertiary-level government hospital. Thus, the findings may differ in other settings. The bias due to the Hawthorne effect cannot be ignored as the staff was aware of being watched in most of the situations.

Recommendations

Effective supply chain management, appropriate infrastructure, and multimodal training programs for all HCWs are essential, along with strict enforcement of hospital waste policy. The practices mentioned above are to be conducted sustainably to make them impactful.

## Conclusions

The challenges faced in BMW practices in the study setting were identified and addressed. The interventions showed improvement in the BMW management practices of staff. The disparity between knowledge and practical skills in managing hospital-generated wastes among HCWs was evident and effectively tackled through targeted training sessions.

However, given the turnover among HCWs within the institute, sustaining this improvement demands ongoing evidence-based training and development initiatives in the infrastructure and supply chain. Embracing customized training modalities is more essential than ever, as they catalyze enriching knowledge and foster best practices in BMW handling and management throughout the hospital.
